# AMPK Regulates Circadian Rhythms in a Tissue- and Isoform-Specific Manner

**DOI:** 10.1371/journal.pone.0018450

**Published:** 2011-03-31

**Authors:** Jee-Hyun Um, Julie S. Pendergast, Danielle A. Springer, Marc Foretz, Benoit Viollet, Alexandra Brown, Myung K. Kim, Shin Yamazaki, Jay H. Chung

**Affiliations:** 1 Laboratory of Obesity and Aging Research, Genetics and Developmental Biology Center, National Heart Lung and Blood Institute, National Institutes of Health, Bethesda, Maryland, United States of America; 2 Department of Biological Sciences, Vanderbilt University, Nashville, Tennessee, United States of America; 3 Mouse Phenotyping Core Facility, National Heart Lung and Blood Institute, National Institutes of Health, Bethesda, Maryland, United States of America; 4 Institut Cochin, Université Paris Descartes, CNRS (UMR 8104), Paris, France; 5 INSERM, U567, Paris, France; Yale School of Medicine, United States of America

## Abstract

**Background:**

AMP protein kinase (AMPK) plays an important role in food intake and energy metabolism, which are synchronized to the light-dark cycle. *In vitro*, AMPK affects the circadian rhythm by regulating at least two clock components, CKIα and CRY1, via direct phosphorylation. However, it is not known whether the catalytic activity of AMPK actually regulates circadian rhythm *in vivo*.

**Methodology/Principal Finding:**

The catalytic subunit of AMPK has two isoforms: α1 and α2. We investigate the circadian rhythm of behavior, physiology and gene expression in AMPKα1−/− and AMPKα2−/− mice. We found that both α1−/− and α2−/− mice are able to maintain a circadian rhythm of activity in dark-dark (DD) cycle, but α1−/− mice have a shorter circadian period whereas α2−/− mice showed a tendency toward a slightly longer circadian period. Furthermore, the circadian rhythm of body temperature was dampened in α1−/− mice, but not in α2−/− mice. The circadian pattern of core clock gene expression was severely disrupted in fat in α1−/− mice, but it was severely disrupted in the heart and skeletal muscle of α2−/− mice. Interestingly, other genes that showed circadian pattern of expression were dysreguated in both α1−/− and α2−/− mice. The circadian rhythm of nicotinamide phosphoryl-transferase (NAMPT) activity, which converts nicotinamide (NAM) to NAD^+^, is an important regulator of the circadian clock. We found that the NAMPT rhythm was absent in AMPK-deficient tissues and cells.

**Conclusion/Significance:**

This study demonstrates that the catalytic activity of AMPK regulates circadian rhythm of behavior, energy metabolism and gene expression in isoform- and tissue-specific manners.

## Introduction

Most organisms exhibit physiological and behavioral rhythms that are controlled by the circadian clock in coordination with the light-dark cycle of the environment [1]. The self-sustained circadian clock consists of autoregulated transcriptional/translational feedback loops of clock genes and their protein products [1]. In mammals, the master circadian clock is located in the hypothalamic suprachiasmatic nuclei (SCN). This master clock is set by light, drives the circadian rhythm of behavior and synchronizes the peripheral clocks [2], [3], [4], [5]. The peripheral clocks in non-light-sensitive organs can also be entrained by other stimuli such as daily feeding [6], [7].

Food intake and energy metabolism are closely linked to the circadian clock. In mice, high fat diet changes the period of the locomotor activity rhythm and disrupts the expression and cycling of circadian clock genes [8]. Also, a number of transcriptional regulators that are primarily involved in metabolic regulation have been shown to play a role in clock function. In peroxisome proliferator-activated receptor α (PPARα)-deficient mice [9], [10], temporally restricted feeding caused a prolonged phase shift of clock gene expression and PPARα responsive genes [11]. Deletion of a related PPAR, PPARγ [12], [13], [14], in endothelial cells blunted the cardiovascular rhythm [15]. The expression of nicotinamide phosphoryl-transferase (NAMPT), which catalyzes NAD^+^ biosynthesis from nicotinamide (NAM), and the levels of NAD^+^ follow a circadian rhythm [16], [17]. The NAD^+^-dependent deacetylase SIRT1, which is activated by energy-deprivation and mediates a diverse array of stress responses [18], [19], regulates the circadian clock by deacetylating PER2 and modulating the activity of CLOCK:BMAL1 complex [20], [21]. One of the targets of SIRT1 is PPARγ coactivator-1α (PGC-1α), the master regulator of mitochondrial biogenesis [22], [23]. Recently, PGC-1α has also been shown to regulate the circadian rhythm by stimulating the expression of the clock gene *Bmal1* [24]. Conversely, the disruption of the circadian clock can lead to metabolic dysregulation. *Clock* mutant mice [25] and *Per2−/−* mice [26] have a disrupted diurnal feeding rhythm and as a result, overeat during the inactive period, leading to obesity.

AMP protein kinase (AMPK) functions as a fuel gauge by sensing increased AMP/ATP ratio [27]. AMPK, when activated by conditions that deplete energy such as hypoxia, ischemia, glucose deprivation and exercise switches on catabolic pathways to generate ATP and to suppress ATP consuming processes. In the hypothalamus, AMPK activity stimulates food intake [28], [29], [30] and in the periphery, AMPK activity stimulates fatty acid uptake and oxidation in addition to glucose uptake [31]. The catalytic subunit of AMPK has two isoforms, α1 and α2, which have different tissue expression patterns. Muscle expresses predominantly α2 isoform [32], whereas fat and brain express predominantly α1 isoform [33]–[34] and liver expresses both α1 and α2 isoforms [35]. Mice deficient in either AMPKα1 [36] or AMPKα2 [37] are viable, but mice deficient in both α1 and α2 are not viable, indicating that the two isoforms have partially redundant functions.

Recently, we and others have shown that AMPK regulates circadian rhythms *in vitro*. AMPK directly phosphorylates and activates the clock component CKIε, which leads to PER2 degradation and a phase advance in the circadian expression of clock genes [38]. AMPK also regulates circadian rhythm by phosphorylating the clock component CRY1 and decreasing its stability [39]. In addition, diurnal shift in energy utilization is blunted in mice deficient in one of the isoforms of the AMP binding subunits (γ3) of AMPK [40].

Although the two α isoforms of AMPK have partially redundant functions, their expression patterns are different and as a result, the two α isoforms may have different functions at the whole-organism level. In this study, we investigate how the two α isoforms of AMPK regulate circadian rhythms by investigating the circadian rhythms of behavior, physiology and gene expression in AMPKα1−/− and AMPKα2−/− mice.

## Results

### AMPK activity has a diurnal rhythm in the hypothalamus

**Figure 1:**
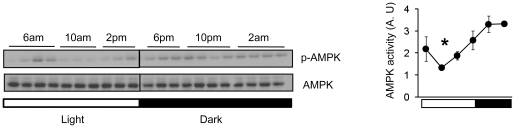
Circadian oscillation of AMPK activity in hypothalamus across the 24 hr light-dark cycle. Left, A representative Western blot showing the phosphorylation level of AMPK (T172) in extracts from mouse hypothalamus which were harvested at 4 hr intervals in a 12h light:12h dark (LD) cycle. The entire hypothalamus from 3 months old male C57BL/6J mice was used for this experiment. Right, Quantification of phosphorylated AMPKα (T172) is shown in arbitrary units (n = 3–4 per time point). Average AMPK activity (arbitrary unit) was calculated by densitometric quantification of phosphorylated proteins normalized to total proteins. Lights-on (6am; light) is indicated by a white bar and lights-off (6pm; dark) is indicated by a black bar. Results are means ± S.E. * P<0.05 between 10 am and 10 pm.

The master pacemaker for rhythmic behavior is located in the suprachiasmatic nucleus (SCN) in the hypothalamus [41], [42], [43], [44]. Although AMPK activity in the hypothalamus is known to stimulate food intake [28], [29], [30], it is not known whether it has a diurnal rhythm in the hypothalamus. We examined AMPK activity by visualizing phosphorylation of T172 in the catalytic subunit of AMPK, in the hypothalamus of C57BL/6J mice during the 24 hr light-dark cycle (12 h light:12 h dark). As shown in Fig. 1, T172 phosphorylation, which reflects the activity of AMPK, displayed a rhythmic pattern, with the peak occurring 4 hr into the dark (active) phase and the nadir occurring 4 hr into the light (inactive) phase. Thus, hypothalamic AMPK activity is higher during the active phase when food intake is highest. We note that the hypothalamus is a heterogeneous brain region, and different nuclei in the hypothalamus (e.g. SCN, Arcuate Nucleus, PVN, etc.) may have different phases of peak AMPK activity than the whole hypothalamus.

### Cell autonomous role of AMPK in circadian rhythm generation

**Figure 2:**
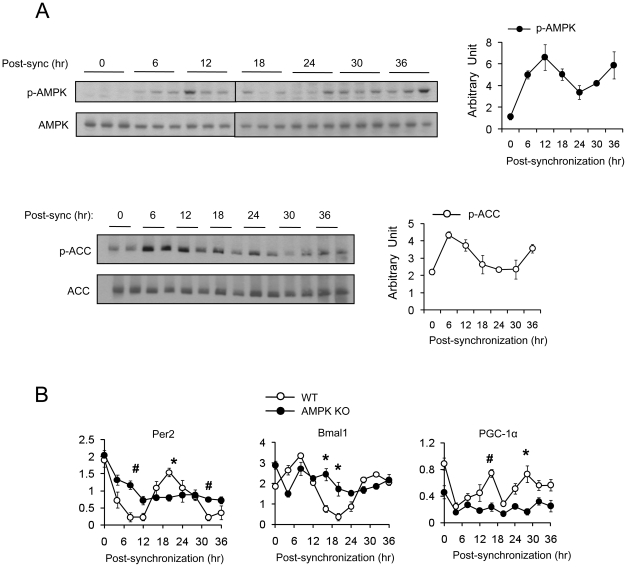
Rhythmic expression of AMPK activity is cell autonomous. (A) WT mefs were synchronized by forskolin and harvested at the indicated time point. Phosphorylated AMPKα (T172) and phosphorylated-ACC (S79) were assessed by Western blot. Average AMPK and ACC activity (arbitrary unit) were calculated by densitometric quantification of phosphorylated proteins normalized to total proteins. Experiments were repeated at least three times. (B) Expression level (arbitrary units) of mPer2, Bmal1 and PGC-1α in WT and AMPKα1/α2 double knockout (AMPK KO) mefs after synchronization with forskolin. Results are means ± S.E. * P<0.05, # P<0.001between WT and AMPK KO mefs.

In live animals, the AMPK activity in the hypothalamus may be influenced by behavioral, physiological or metabolic fluctuations during the 24 hr cycle. Therefore, whether the AMPK cycle is cell-autonomous or not cannot be addressed in live animals. To test whether the cyclic pattern of AMPK activity is cell autonomous, we examined T172 phosphorylation over a period 36 hr in wild type murine embryo fibroblasts (mefs) after synchronizing with forskolin [45]. AMPK phosphorylation in mefs exhibited an oscillatory pattern with a 24 hr period, with the maximal and minimal phosphorylation occurring approximately 12 hr and 24 hr after foskolin treatment, respectively (Fig 2A). AMPK-mediated phophorylation of ACC1 (S79) [46], [47] also exhibited an oscillatory pattern that closely resembled the AMPK phosphorylation pattern. These findings indicate that AMPK activity has cell-autonomous circadian rhythm.

### AMPK is essential for circadian rhythm generation in mefs

In order to determine whether AMPK is required for circadian rhythm, we measured the mRNA levels of circadian genes *Per2*, *Bmal1* and *PGC-1α* in WT and AMPKα1/α2−/− mefs after forskolin synchronization. As shown in Fig. 2B, the oscillatory pattern of *Per2*, *Bmal1* and *PGC-1α* expression was not present in AMPKα1/α2−/− mefs. These results indicate that not only is AMPK activity regulated by the circadian rhythm, but that it is also essential for circadian rhythm generation.

### AMPKα1−/− and AMPKα2−/− mice have an altered free-running period and feeding rhythm

**Figure 3:**
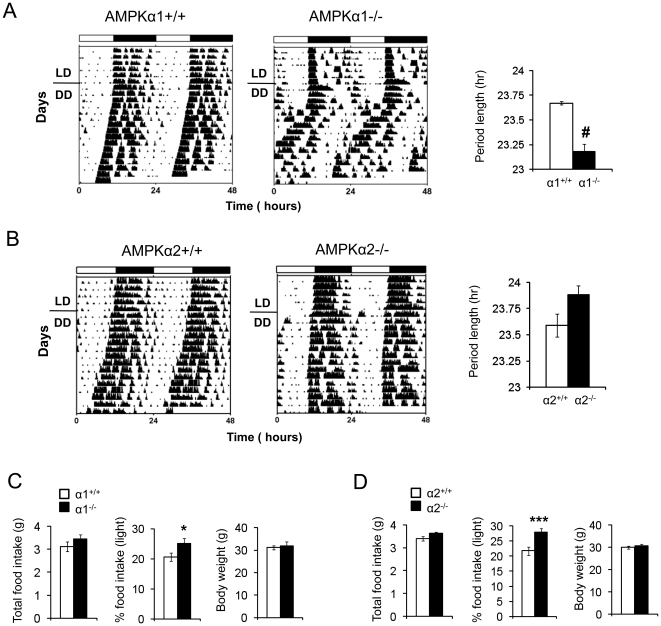
Altered free-running period in AMPKα-deficient mice. (A–B) The activity rhythm was monitored by wheel running under light:dark cycles 12 hr:12 hr (LD) or under constant darkness (DD). Activity records of representative AMPKα1−/− and AMPKα2 −/− mice and their wild-type littermates are shown in double plotted actograms. Each horizontal line represents a 48 hr period and the vertical bars represent wheel running in 10-minute bins (n = 5–6). # P<0.0001, between AMPKα1+/+ and AMPKα1−/− mice. The free-running period was determined by using the χ2 periodogram for days 1–14 in DD. The periods of AMPKα1−/−, AMPKα2−/− and their wild type littermate mice are shown in the right panel. (C,D) Total 24 hr food intake, % of food intake during the light period and body weight of AMPKα1−/− and AMPKα2−/− and their wild-type littermates (n = 10). Results are means ± S.E. * P<0.05 between AMPKα1+/+ and AMPKα1−/−mice. *** P<0.001 between AMPKα2+/+ and AMPKα2−/− mice.

The SCN clock drives the circadian rhythm of locomotor activity. Since AMPK is essential for circadian rhythm generation in mefs, we investigated whether it is important for circadian rhythm generation in the SCN. We monitored the free-running locomotor activity in AMPKα1−/− and AMPKα2−/− mice and their littermate controls. For this purpose, we used two different environmental conditions: A light-dark (LD) cycle, in which mice are exposed to 12 hr of light (6 am–6 pm) and 12 hr of darkness (6 pm–6 am) and a dark-dark (DD) cycle, in which mice are exposed to constant darkness. The presence of circadian rhythmicity in DD is indicative of a functioning internal clock. As shown in Fig. 3A and B, both AMPKα1−/− and AMPKα2−/− mice exhibited persistent circadian rhythmicity in DD, and the amplitudes of locomotor activity were similar to that of the WT littermates. The presence of circadian rhythmicity in the absence of daily light entrainment (i.e. DD) indicates that the SCN clock in AMPKα1−/− and AMPKα2−/− mice is largely intact. However in the absence of light entrainment, the free running period of AMPKα1−/− mice was shorter than that of AMPKα1+/+ littermates (23.2 hr vs. 23.7 hr, P = 0.0003). In contrast, the free running period of AMPKα2-/− was longer than that of AMPKα2+/+ littermates (23.9 hr vs. 23.6 hr), but this did not reach statistical significance (P = 0.07). The period lengths of C57BL/6J, AMPKα1+/+ and AMPKα2+/+ mice were nearly identical (Figure S1).

Since AMPK activity in the hypothalamus stimulates food intake [28], [29], [30], it may play a role in the diurnal rhythm of food intake. To test this, we measured food intake of AMPKα1−/−, AMPKα2−/− and their littermate controls during the light and dark phases. The total food intake and body weight for AMPKα1−/−, AMPKα2−/− and their littermate controls was the same (Fig. 3 C, D). As nocturnal animals, mice consume most of their food during the dark phase. Both AMPKα1−/− and AMPKα2−/− mice ate more food during the light phase than their wild-type littermates (20.6% vs. 25.1% for the AMPKα1 pair and 21.8% vs. 28% for the AMPKα2 pair) (Fig. 3C), indicating that AMPK-deficient mice have blunted feeding rhythm.

### AMPK is important for body temperature rhythm

**Figure 4:**
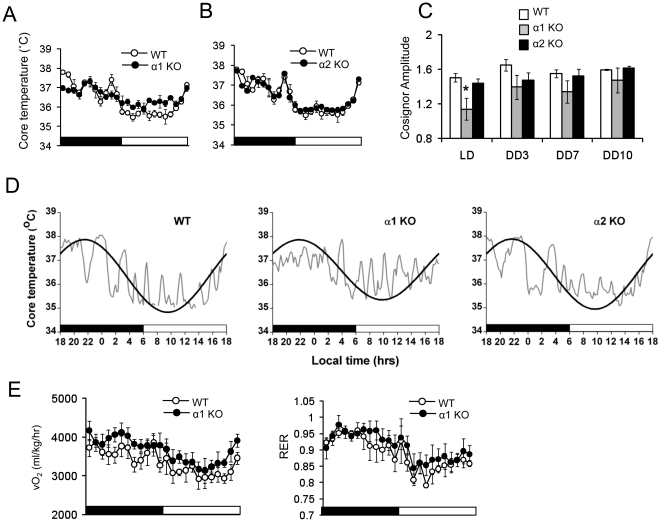
Disruption of circadian physiology in AMPKα-deficient mice. (A,B) Core body temperature was measured by telemetry. AMPKα1−/−, AMPKα2−/− and WT mice were monitored in LD for 7 days followed by DD for 14 days. Representative data (mean ± SE) is LD day 7. Lights on is indicated by a white bar and lights off is indicated by a black bar. The same WT data is plotted in A and B. (C) Amplitude calculated from cosinor analysis of WT, AMPKα1−/− and AMPKα2−/− mice in LD, DD day 3, 7, 10. Results are expressed as mean ± S.E. * P<0.05 between WT mice versus AMPKα1 deficient mice (n = 3–5 for each genotype). (D) Cosinor analysis of core temperature for WT, AMPKα1−/− and AMPKα2−/− mice. The cosine curve (black line) is superimposed on core temperature raw data (gray line). A representative plot is shown for each genotype (E) VO_2_ (left) and RER (right) in AMPKα1−/− and WT mice. Average data is shown under LD on day 7. Light on is indicated by a white bar and light off is indicated by a black bar (n = 4 for each genotype).

We investigated whether AMPK is important for the circadian rhythmicity of metabolic parameters such as body temperature in light-dark (LD) as well as in constant darkness (DD) and oxygen consumption (VO_2_) (Fig 4). Compared to WT mice, AMPKα1−/− mice clearly had dampened circadian rhythm of core temperature (Fig. 4A). However, circadian rhythm of core temperature of AMPKα2−/− appeared to be very similar to that of WT mice (Fig. 4B). To better quantify the amplitude of the core temperature rhythm, we performed cosinor analysis. A representative cosinor plot for each genotype in light-dark (LD) is shown in Fig. 4D. The calculations of the amplitude of the cosine curves indicate that AMPKα1−/− had lower amplitude in LD than either WT or AMPKα2−/− mice (Fig. 4C). The amplitude of the cosine curves of AMPKα1−/− mice was also lower in DD, but the difference did not reach statistical significance.

We then compared VO_2_ of WT and AMPKα1−/− mice in LD. Although AMPKα1−/− mice tended to have higher VO_2_ than WT mice, they had similar amplitude of the circadian rhythm of VO_2_ (Fig. 4E). To assess energy utilization, we measured respiratory exchange ratio (RER) in AMPKα1−/− mice and WT mice. There was clear diurnal shift for energy utilization in both AMPKα1−/− mice. AMPKα1−/− mice showed a tendency toward the blunting of the shift, but it was not statistically significant. Taken together, these results indicate that AMPKα1 is required for generating normal amplitudes of core temperature rhythm, but not VO_2_ or RER rhythms.

### Expression patterns of Clock genes in peripheral tissues in AMPKα deficient mice

**Figure 5:**
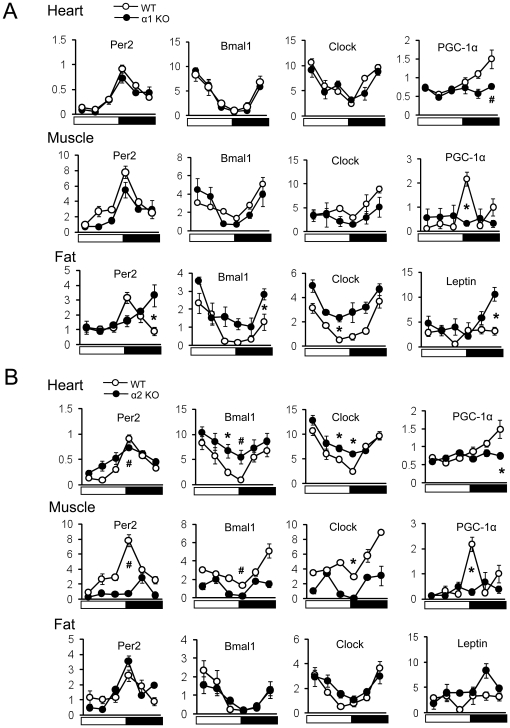
Clock and clock related gene expression in heart, skeletal muscle and fat in (A) AMPKα1−/−, (B) AMPKα2−/− and WT mice. mRNA levels of mPer2, Bmal1, Clock, PGC-1α and leptin were measured by real-time PCR. The relative levels of mRNA are presented in arbitrary units. Light on is indicated by a white bar and light off is indicated by a black bar. The same WT data is plotted in A and B. Results are mean ± S.E. * P<0.05, # P<0.001 between WT versus AMPKα deficient mice (n = 4–5 for each time point).

The circadian clock in peripheral tissues is self-sustained and can be entrained by food [6], [7], [31], [48]. To further explore the role of AMPK in peripheral clock function, we examined the daily expression profile of circadian genes in the heart, gastrocnemius muscle and epididymal white fat in AMPKα1−/− and AMPKα2−/− mice. As shown in Fig. 5, the expression pattern of the core clock genes (*Per2, Bmal1, and Clock*) exhibited a 24 hr rhythmicity in the heart, skeletal muscle and fat of WT mice, consistent with previous observations [49], [50]. The heart and skeletal muscle express predominantly AMPKα2 and very little AMPKα1. On the other hand, fat expresses predominantly AMPKα1 and very little AMPKα2. Consistent with this expression pattern of the two isoforms, the cyclic expression pattern of the core clock genes was preserved in the heart and skeletal muscle of AMPKα1−/− mice (Fig. 5A), but they were significantly blunted in AMPKα2−/− mice (Fig. 5B). Also as expected, the cyclic expression pattern of clock genes was blunted in AMPKα1−/− fat (Fig. 5A) but not in AMPKα2−/− fat (Fig. 5B). Surprisingly, the expression patterns of *PGC-1α and leptin* did not fit this pattern. The cyclic expression pattern of *PGC-1α* was disrupted in the heart and skeletal muscle of both AMPKα1−/− and AMPKα2−/− mice. Similarly, the cyclic expression pattern of *leptin* was disrupted in the fat of both AMPKα1−/− and AMPKα2−/− mice. Therefore, although the role of each isoform of AMPK in generating the cyclic expression pattern of core clock genes correlated with their relative abundance in peripheral tissues, the cyclic expression pattern of *PGC-1α and leptin* did not.

### Circadian oscillation of *Nampt* mRNA and NAD+ production requires AMPK

**Figure 6:**
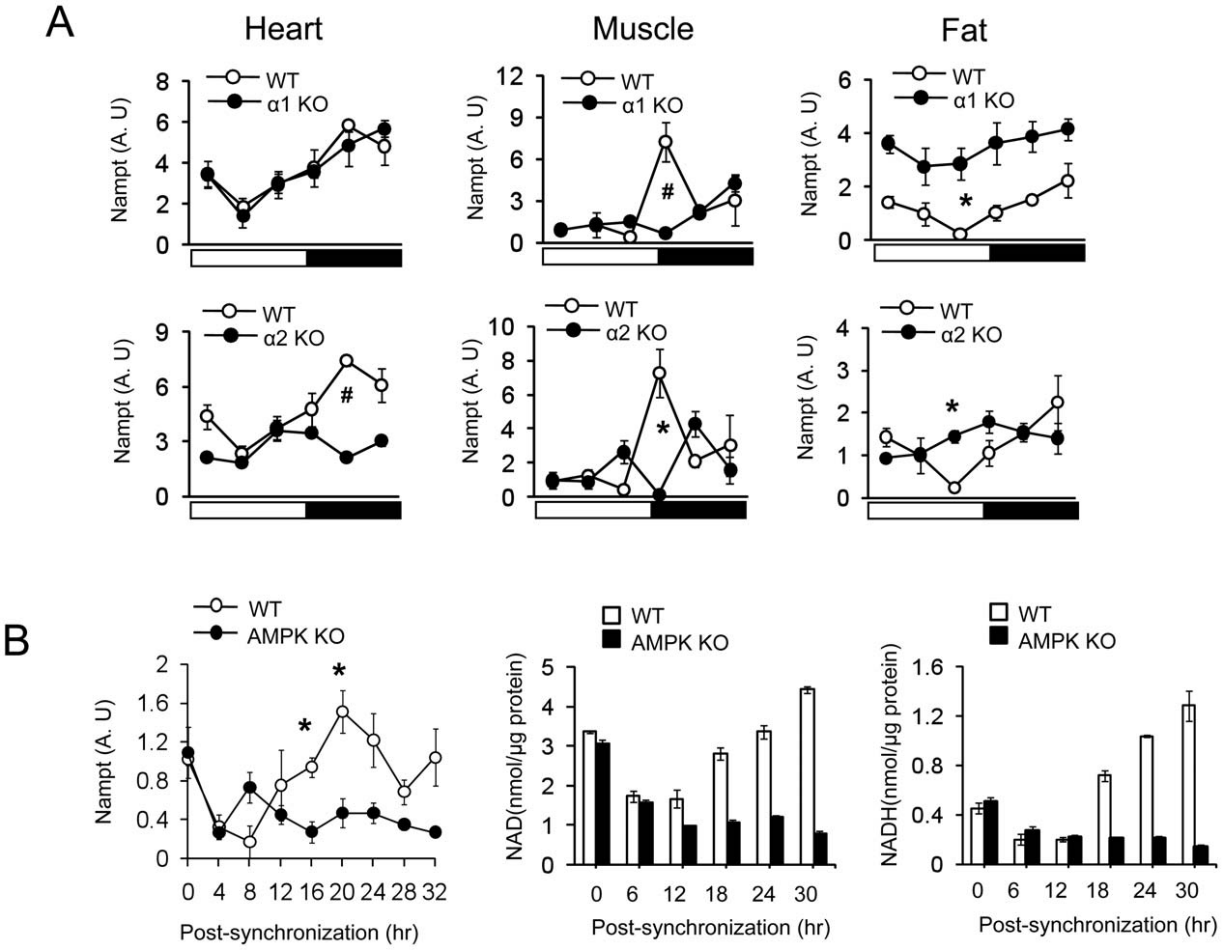
Circadian oscillation of NAD+ and Nampt gene expression requires AMPK. (A) Nampt gene expression patterns of heart, skeletal muscle and fat tissue during 24 hr for AMPKα1−/−, AMPKα2 −/− and WT mice. The relative levels of mRNA are presented in arbitrary units. Results are expressed as mean ± S.E., * P<0.05, # P<0.001 between WT and AMPKα deficient mice. (n = 4–5 for each time point). The same WT data is plotted for α1 KO and α2 KO. (B) Nampt gene expression (arbitrary units) and cellular NAD+ and NADH level in WT and AMPKα1/α2 KO mefs synchronized with forskolin. Results are expressed as mean ± S.E., * P<0.05, between WT mefs and AMPKα1/α2 KO mefs (n = 3).

AMPK has been shown to increase the expression of *Nampt* and the product of its enzymatic reaction, NAD+ [51], [52]. Since the circadian oscillation of NAD+ levels promotes circadian rhythm generation via SIRT1 [17], we investigated whether AMPK is required for circadian oscillation of *Nampt* mRNA. As expected, *Nampt* mRNA levels displayed robust circadian oscillation in heart, skeletal muscle and fat in WT mice. However, circadian oscillation of *Nampt* mRNA was intact in the heart but not in skeletal muscle or fat of AMPKα1−/− mice (Fig. 6A). On the other hand, circadian oscillation of *Nampt* mRNA was significantly blunted in all three tissues of AMPKα2−/− mice. Therefore, as was the case with *PGC-1α* mRNA and *leptin* mRNA (Fig. 5), the circadian oscillation of *Nampt* mRNA expression in skeletal muscle and fat required both isoforms. To investigate whether circadian oscillation of *Nampt* mRNA is cell-autonomous, we measured *Nampt* mRNA levels in WT and AMPKα1/α2−/− mefs after forskolin synchronization. *Nampt* mRNA levels displayed circadian oscillation with the peak occurring 20 hrs after synchronization in WT mefs but not in AMPKα1/α2−/− mefs (Fig. 6B). Consistent with this, intracellular NAD+ and NADH levels displayed circadian oscillation in WT mefs but not in AMPKα1/α2−/− mefs. Taken together, these results indicate that AMPK promotes circadian rhythm in part by generating the circadian oscillation of NAD+ production.

## Discussion

AMPK regulates energy intake and expenditure to maintain cellular and whole body energy metabolism, which is coupled with daily light-dark cycles. To understand the intrinsic role of AMPK on circadian rhythms *in vivo*, we studied the circadian behavior and physiology of mice deficient in AMPKα1 or AMPKα2. We found that the circadian behavior of feeding and free-running period of AMPKα1−/− mice and AMPK α2−/− mice were dysregulated. Circadian rhythms of core temperature were dysregulated in AMPKα1−/− mice but not in AMPK α2−/− mice. There was no difference in the circadian rhythm of VO_2_ between WT mice and AMPKα1−/− mice.

In the hypothalamus, AMPK is a master regulator of food intake. Fasting increases AMPK activity and stimulates food intake, while refeeding suppress it [28], [29]. It is intriguing that hypothalamic AMPK activity has a diurnal oscillation that peaks during the dark period when mice are active and eating. Thus, the timing of hypothalamic AMPK activity correlates with the timing of appetite.

We and others have previously demonstrated the effects of the molecular mechanisms of AMPK on circadian clockwork circuitry. For example AMPK induces a phase advance of circadian expression of clock genes by degrading PER2 through phosphorylating Casein kinase Iε Ser389 [38] and AMPK contributes to metabolic entrainment of peripheral clocks by phosphorylating and destabilizing CRY1 [39]. In addition, the circadian rhythm of clock genes is absent in AMPKα1/α2−/− mefs.

Thus it is possible that AMPK is a critical component or output of the central clock in the SCN hypothalamus. It is interesting to note that AMPKα1−/− mice exhibit a shortened period while AMPKα2−/− mice tended to exhibit a longer period suggesting that the α1 and α2 isoform of AMPK may have distinct roles in regulation of circadian period. Consistent with this, it has been observed that AMPKα2 activity is decreased in response to leptin injection in the hypothalamus but AMPKα1 activity is unchanged [28]. Further study is needed to evaluate the regulation of AMPK specifically on SCN and its neuronal network in hypothalamus.

In the peripheral tissue, AMPK controls energy metabolism by regulating the activity or expression of metabolic genes [31]. We found that AMPK regulates expression of peripheral clock genes in an isoform- and tissue-specific manner. One surprising discovery in this study was that the cyclic expression pattern of *PGC-1α, leptin and Nampt* required the presence of both AMPK isoforms even though only one isoform was predominantly expressed in the tissues we studied: α1 in fat and α2 in the heart and skeletal muscle (Fig. 5). There may be several explanations for this that are not mutually exclusive. One explanation is that the two isoforms have non-overlapping functions even though the expression level of one isoform is significantly lower than the other. For example, in skeletal muscle, both AMPKα1 and α2 activities increased during treadmill running [53] but only AMPKα2 is required for glucose uptake after AICAR stimulation whereas AMPKα1 activation is required for glucose uptake after twitch contraction [36], [54]. Another explanation is that the cyclic expression pattern of *PGC-1α, leptin and Nampt* is more sensitive to AMPK dosage than that of the core clock genes. Finally, the cyclic expression pattern of *PGC-1α, leptin and Nampt* may also depend on extracellular signals. Circadian gene expression in peripheral tissues is intimately connected to feeding and the nutrient state [6], [7], [8], [48]. Since the expression of leptin [55], PGC-1α [56] and Nampt [57] is regulated by food intake, and the feeding rhythm is blunted in both AMPKα1−/− and AMPKα2−/− mice (Fig. 3C), it is possible that the disruption of the expression pattern of *leptin, PGC-1α and Nampt* mRNA may, at least in part, have resulted from feeding rhythm disruption. In addition, there may be cross-talks between fat and the heart or skeletal muscle that may be important for the cyclic expression pattern of *leptin and PGC-1α* in which case the AMPK deficiency in one tissue may affect the expression pattern of *leptin or PGC-1α* in another tissue.

The activity of SIRT1 and the NAD+ salvage pathway regulate the circadian rhythm [20], [21]. NAD+ level and Nampt, a rate limiting enzyme mediating NAD+ biosynthesis, cycles with a 24-hour rhythm [17]. Recent studies showed that activation of AMPK enhances SIRT1 activity by increasing *Nampt* expression and intracellular NAD+ levels, which induces deacetylation of SIRT1 targets such as PGC-1α [52], [58]. Consistent with this we found that rhythmic expression of *Nampt and PGC-1α* was abolished in both AMPKα1−/− and AMPKα2−/− mice. It has been shown that PGC-1α is important for circadian rhythm generation in skeletal muscle and liver [24] and is also an AMPK substrate. Our results indicate that the circadian regulation of the Nampt-SIRT1-PGC-1α pathway is at least partially dependent on AMPK *in vivo*.

In summary, the role of AMPK in generating free-running period, metabolic rhythms and clock gene expression in peripheral tissues is tissue- and isoform-specific. Furthermore AMPK is required for the cycling of NAD+ level and circadian expression of *Nampt and PGC-1α*. Thus, we demonstrated the importance of AMPK in the circadian rhythms of behavior, energy metabolism and gene expression at the whole-organism level. Except for the gene expression changes (Fig. 5 and Fig. 6), the degree to which the circadian rhythm is disrupted in these AMPK-deficient mice is rather modest. However, this is not surprising since only one of the two isoforms is missing in each AMPK-deficient mouse, and it is well known that there is some functional redundancy between the two isoforms. Moreover, as it has been shown in skeletal muscle [36], deletion of one isoform can lead to a compensatory increase in the expression of the other isoform. Thus, conditional-knockout of both isoforms will be needed to fully demonstrate the role of AMPK in circadian rhythms.

## Materials and Methods

### Animals

Generation of AMPKα1−/− [36] and AMPKα2−/− [37] mice was previously described. AMPKα1−/− and AMPKα2−/− mice and their littermates were generated by backcrossing AMPKα1−/− and AMPKα2−/− mice to C57BL/6J mice (Jackson Laboratory, Bar Harbor, ME) for 6 generations followed by brother-sister mating. We used age-matched male mice for all our studies. For period length measurements and circadian rhythm of food intake studies, we used wile-type littermates as controls. For real-time PCR, indirect calorimetry and body temperature studies, we used C57BL/6J mice as wild-type controls. The mice were bred and group-housed in the animal facility in a 12 h-light/12 h-dark cycle (12L:12D) and provided food and water *ad libitum*. All experiments were approved by NHLBI ACUC (Animal Care and Use Committee).

### Body weight and food intake measurements

Body weight and food intake were monitored during 5 days in four months old male AMPKα1−/−, AMPKα2−/− and their wild-type littermates exposed to a 12 hr:12 hr LD cycle.

### Mouse wheel running activity

Voluntary mouse activity was measured by activity wheel running. Two months old male AMPKα1−/− and AMPKα2−/− mice and their wild-type littermates were individually placed into cages with a running wheel and allowed to acclimate for one week prior to the experiment. Activity was measured with activity wheel counters (model 86061), an Animal Wheel Monitor Starter Interface (model 86056) and AWM software provided by Layfayette Instrument Company, Inc (Layfaytte, IN). Data were downloaded at 10 minute intervals during a 12 hr light/12 hr dark (LD) cycle for 10 days followed by a 24 hr constant darkness (DD) cycle for 30 days. Clock Lab (Actimetrics) Data Analysis software was used to produce the double-plotted actogram and period.

### Indirect calorimetry

Metabolic rate (VO_2_) was determined by Oxymax chambers (Columbus Instruments). Energy expenditure was recorded by calculating the average energy expenditure for each 10 min time point during one week of LD 12 hr:12 hr cycle followed by three weeks of DD 12 hr:12 hr cycle. A few time points (<14 out of 1152) were found to have negative values. These outlier values were removed before calculation. Respiratory exchange ratio (RER) was calculated as the ratio between the CO_2_ production and the O_2_ consumption.

### Body temperature

For internal body temperature monitoring, mice were implanted with Data Science International (DSI) ETA-F20 transmitters. The transmitter was placed in the abdominal cavity and the ECG leads were sutured to the chest wall in a Lead II position. Animals were allowed to recover for two weeks prior to the initiation of monitoring using telemetric receivers. Body temperature was recorded under LD 12 hr:12 hr for one week followed by DD 12 hr:12 hr for three more weeks. Data were analyzed using DSI Ponemah software.

### Cosinor Analysis

To analyze the amplitude of the core temperature rhythms, cosinor analysis was performed using Circadian Physiology software [59]. Cosine curves were fit to the data using a fixed 24-hr period. Prior to data analysis, data points showing a temperature below 35°C (<13 out of 7000 data points) were removed from each dataset as outlier data points.

### Cell culture

AMPKα1/α2 double knockout and WT mouse embryo fibroblasts (mefs) [60] were maintained in high glucose Dulbecco's Modified Eagle Medium (DMEM) with 10% fetal bovine serum. Confluent cells were synchronized with 10 µM of forskolin (Calbiochem) treatment for 30 min.

### Immunoblotting

Cells were lysed in RIPA buffer and subjected to Western blot. Dissected hypothalami from three months old male C57LB/6J mice were immediately frozen in liquid nitrogen and homogenized in lysis buffer [61]. Phosphorylation of AMPKα and ACC were determined by electrophoresis on 4–12% gradient SDS-polyacrylamide gel followed by anti-phospho AMPKα (T172) and anti-phospho ACC (S79) antibodies from Cell Signaling.

### Real-time PCR

Heart, gastrocnemius muscle and epididymal white fat were isolated every 4 hr for a total 24 hr (mice maintained in 12L:12D) and pulverized in liquid nitrogen. Total RNA was isolated using the TRIzol reagent extraction kit (Invitrogen), according to manufacturer's instructions. RNA was subsequently reverse-transcribed to cDNA by using the high capacity cDNA archive kit (ABI). The mRNA levels were measured by real time PCR using the TaqMan Gene Expression system and the ABI PRISMTM 7900HT Sequence Detection System (Applied Biosystem).

### NAD+ and NADH level measurement

The NAD+ and NADH levels were measured from whole cell extracts of WT mefs and AMPK α1/α2 double knockout mefs synchronized by with forskolin, by using the Biovision NAD/NADH Quantification kit according to the manufacturer's instruction.

### Statistical analysis

Independent *t* tests (two-tailed) were used to compare two groups. For comparison between three groups, one-way ANOVA followed by Bonferroni post-test was used. Significance was accepted at P<0.05 unless indicated otherwise. Results are expressed as the mean ±SEM

## Supporting Information

Figure S1 **Comparison of the free-running period of wild-type controls.** The free-running period of C57BL/6J (n = 5), AMPKα1+/+ (n = 5) and AMPKα2 +/+ (n = 7) mice are shown. The free-running period was determined by using the χ2 periodogram for days 1–14 in DD. Result is expressed as means ± SEM.
(TIF)

## References

[1] Reppert SM, Weaver DR (2002). Coordination of circadian timing in mammals.. Nature.

[2] Ibuka N, Inouye SI, Kawamura H (1977). Analysis of sleep-wakefulness rhythms in male rats after suprachiasmatic nucleus lesions and ocular enucleation.. Brain Res.

[3] Ibuka N, Nihonmatsu I, Sekiguchi S (1980). Sleep-wakefulness rhythms in mice after suprachiasmatic nucleus lesions.. Waking Sleeping.

[4] Welsh D, Richardson GS, Dement WC (1988). Effect of running wheel availability on circadian patterns of sleep and wakefulness in mice.. Physiol Behav.

[5] Lowrey PL, Takahashi JS (2004). Mammalian circadian biology: elucidating genome-wide levels of temporal organization.. Annu Rev Genomics Hum Genet.

[6] Stokkan KA, Yamazaki S, Tei H, Sakaki Y, Menaker M (2001). Entrainment of the circadian clock in the liver by feeding.. Science.

[7] Damiola F, Le Minh N, Preitner N, Kornmann B, Fleury-Olela F (2000). Restricted feeding uncouples circadian oscillators in peripheral tissues from the central pacemaker in the suprachiasmatic nucleus.. Genes Dev.

[8] Kohsaka A, Laposky AD, Ramsey KM, Estrada C, Joshu C (2007). High-fat diet disrupts behavioral and molecular circadian rhythms in mice.. Cell Metab.

[9] Leone TC, Weinheimer CJ, Kelly DP (1999). A critical role for the peroxisome proliferator-activated receptor alpha (PPARalpha) in the cellular fasting response: the PPARalpha-null mouse as a model of fatty acid oxidation disorders.. Proc Natl Acad Sci U S A.

[10] Kersten S, Seydoux J, Peters JM, Gonzalez FJ, Desvergne B (1999). Peroxisome proliferator-activated receptor alpha mediates the adaptive response to fasting.. J Clin Invest.

[11] Goh BC, Wu X, Evans AE, Johnson ML, Hill MR (2007). Food entrainment of circadian gene expression altered in PPARalpha-/- brown fat and heart.. Biochem Biophys Res Commun.

[12] Barak Y, Nelson MC, Ong ES, Jones YZ, Ruiz-Lozano P (1999). PPAR gamma is required for placental, cardiac, and adipose tissue development.. Mol Cell.

[13] Rosen ED, Sarraf P, Troy AE, Bradwin G, Moore K (1999). PPAR gamma is required for the differentiation of adipose tissue in vivo and in vitro.. Mol Cell.

[14] Kubota N, Terauchi Y, Miki H, Tamemoto H, Yamauchi T (1999). PPAR gamma mediates high-fat diet-induced adipocyte hypertrophy and insulin resistance.. Mol Cell.

[15] Wang N, Yang G, Jia Z, Zhang H, Aoyagi T (2008). Vascular PPARgamma controls circadian variation in blood pressure and heart rate through Bmal1.. Cell Metab.

[16] Nakahata Y, Sahar S, Astarita G, Kaluzova M, Sassone-Corsi P (2009). Circadian control of the NAD+ salvage pathway by CLOCK-SIRT1.. Science.

[17] Ramsey KM, Yoshino J, Brace CS, Abrassart D, Kobayashi Y (2009). Circadian clock feedback cycle through NAMPT-mediated NAD+ biosynthesis.. Science.

[18] Bordone L, Guarente L (2005). Calorie restriction, SIRT1 and metabolism: understanding longevity.. Nat Rev Mol Cell Biol.

[19] Dali-Youcef N, Lagouge M, Froelich S, Koehl C, Schoonjans K (2007). Sirtuins: the ‘magnificent seven’, function, metabolism and longevity.. Ann Med.

[20] Asher G, Gatfield D, Stratmann M, Reinke H, Dibner C (2008). SIRT1 regulates circadian clock gene expression through PER2 deacetylation.. Cell.

[21] Nakahata Y, Kaluzova M, Grimaldi B, Sahar S, Hirayama J (2008). The NAD+-dependent deacetylase SIRT1 modulates CLOCK-mediated chromatin remodeling and circadian control.. Cell.

[22] Puigserver P, Wu Z, Park CW, Graves R, Wright M (1998). A cold-inducible coactivator of nuclear receptors linked to adaptive thermogenesis.. Cell.

[23] Wu Z, Puigserver P, Andersson U, Zhang C, Adelmant G (1999). Mechanisms controlling mitochondrial biogenesis and respiration through the thermogenic coactivator PGC-1.. Cell.

[24] Liu C, Li S, Liu T, Borjigin J, Lin JD (2007). Transcriptional coactivator PGC-1alpha integrates the mammalian clock and energy metabolism.. Nature.

[25] Turek FW, Joshu C, Kohsaka A, Lin E, Ivanova G (2005). Obesity and metabolic syndrome in circadian Clock mutant mice.. Science.

[26] Yang S, Liu A, Weidenhammer A, Cooksey RC, McClain D (2009). The role of mPer2 clock gene in glucocorticoid and feeding rhythms.. Endocrinology.

[27] Hardie DG (2007). AMP-activated/SNF1 protein kinases: conserved guardians of cellular energy.. Nat Rev Mol Cell Biol.

[28] Minokoshi Y, Alquier T, Furukawa N, Kim YB, Lee A (2004). AMP-kinase regulates food intake by responding to hormonal and nutrient signals in the hypothalamus.. Nature.

[29] Andersson U, Filipsson K, Abbott CR, Woods A, Smith K (2004). AMP-activated protein kinase plays a role in the control of food intake.. J Biol Chem.

[30] Lopez M, Lage R, Saha AK, Perez-Tilve D, Vazquez MJ (2008). Hypothalamic fatty acid metabolism mediates the orexigenic action of ghrelin.. Cell Metab.

[31] Kahn BB, Alquier T, Carling D, Hardie DG (2005). AMP-activated protein kinase: ancient energy gauge provides clues to modern understanding of metabolism.. Cell Metab.

[32] Stapleton D, Mitchelhill KI, Gao G, Widmer J, Michell BJ (1996). Mammalian AMP-activated protein kinase subfamily.. J Biol Chem.

[33] Lihn AS, Jessen N, Pedersen SB, Lund S, Richelsen B (2004). AICAR stimulates adiponectin and inhibits cytokines in adipose tissue.. Biochem Biophys Res Commun.

[34] Cheung PC, Salt IP, Davies SP, Hardie DG, Carling D (2000). Characterization of AMP-activated protein kinase gamma-subunit isoforms and their role in AMP binding.. Biochem J 346 Pt.

[35] Woods A, Salt I, Scott J, Hardie DG, Carling D (1996). The alpha1 and alpha2 isoforms of the AMP-activated protein kinase have similar activities in rat liver but exhibit differences in substrate specificity in vitro.. FEBS Lett.

[36] Jorgensen SB, Viollet B, Andreelli F, Frosig C, Birk JB (2004). Knockout of the alpha2 but not alpha1 5′-AMP-activated protein kinase isoform abolishes 5-aminoimidazole-4-carboxamide-1-beta-4-ribofuranosidebut not contraction-induced glucose uptake in skeletal muscle.. J Biol Chem.

[37] Viollet B, Andreelli F, Jorgensen SB, Perrin C, Geloen A (2003). The AMP-activated protein kinase alpha2 catalytic subunit controls whole-body insulin sensitivity.. J Clin Invest.

[38] Um JH, Yang S, Yamazaki S, Kang H, Viollet B (2007). Activation of 5′-AMP-activated kinase with diabetes drug metformin induces casein kinase Iepsilon (CKIepsilon)-dependent degradation of clock protein mPer2.. J Biol Chem.

[39] Lamia KA, Sachdeva UM, DiTacchio L, Williams EC, Alvarez JG (2009). AMPK regulates the circadian clock by cryptochrome phosphorylation and degradation.. Science.

[40] Vieira E, Nilsson EC, Nerstedt A, Ormestad M, Long YC (2008). Relationship between AMPK and the transcriptional balance of clock-related genes in skeletal muscle.. Am J Physiol Endocrinol Metab.

[41] Saper CB, Scammell TE, Lu J (2005). Hypothalamic regulation of sleep and circadian rhythms.. Nature.

[42] Weaver DR (1998). The suprachiasmatic nucleus: a 25-year retrospective.. J Biol Rhythms.

[43] Ralph MR, Foster RG, Davis FC, Menaker M (1990). Transplanted suprachiasmatic nucleus determines circadian period.. Science.

[44] Okamura H (2007). Suprachiasmatic nucleus clock time in the mammalian circadian system.. Cold Spring Harb Symp Quant Biol.

[45] Balsalobre A, Marcacci L, Schibler U (2000). Multiple signaling pathways elicit circadian gene expression in cultured Rat-1 fibroblasts.. Curr Biol.

[46] Ha J, Daniel S, Broyles SS, Kim KH (1994). Critical phosphorylation sites for acetyl-CoA carboxylase activity.. J Biol Chem.

[47] Ruderman NB, Saha AK, Vavvas D, Witters LA (1999). Malonyl-CoA, fuel sensing, and insulin resistance.. Am J Physiol.

[48] Yoo SH, Yamazaki S, Lowrey PL, Shimomura K, Ko CH (2004). PERIOD2::LUCIFERASE real-time reporting of circadian dynamics reveals persistent circadian oscillations in mouse peripheral tissues.. Proc Natl Acad Sci U S A.

[49] Yamamoto T, Nakahata Y, Soma H, Akashi M, Mamine T (2004). Transcriptional oscillation of canonical clock genes in mouse peripheral tissues.. BMC Mol Biol.

[50] Ando H, Yanagihara H, Hayashi Y, Obi Y, Tsuruoka S (2005). Rhythmic messenger ribonucleic acid expression of clock genes and adipocytokines in mouse visceral adipose tissue.. Endocrinology.

[51] Fulco M, Cen Y, Zhao P, Hoffman EP, McBurney MW (2008). Glucose restriction inhibits skeletal myoblast differentiation by activating SIRT1 through AMPK-mediated regulation of Nampt.. Dev Cell.

[52] Canto C, Gerhart-Hines Z, Feige JN, Lagouge M, Noriega L (2009). AMPK regulates energy expenditure by modulating NAD+ metabolism and SIRT1 activity.. Nature.

[53] Jorgensen SB, Wojtaszewski JF, Viollet B, Andreelli F, Birk JB (2005). Effects of alpha-AMPK knockout on exercise-induced gene activation in mouse skeletal muscle.. FASEB J.

[54] Jensen TE, Schjerling P, Viollet B, Wojtaszewski JF, Richter EA (2008). AMPK alpha1 activation is required for stimulation of glucose uptake by twitch contraction, but not by H2O2, in mouse skeletal muscle.. PLoS One.

[55] Saladin R, De Vos P, Guerre-Millo M, Leturque A, Girard J (1995). Transient increase in obese gene expression after food intake or insulin administration.. Nature.

[56] Nisoli E, Tonello C, Cardile A, Cozzi V, Bracale R (2005). Calorie restriction promotes mitochondrial biogenesis by inducing the expression of eNOS.. Science.

[57] Arany Z, Lebrasseur N, Morris C, Smith E, Yang W (2007). The transcriptional coactivator PGC-1beta drives the formation of oxidative type IIX fibers in skeletal muscle.. Cell Metab.

[58] Canto C, Jiang LQ, Deshmukh AS, Mataki C, Coste A (2010). Interdependence of AMPK and SIRT1 for metabolic adaptation to fasting and exercise in skeletal muscle.. Cell Metab.

[59] Refinetti R (2006). Circadian physiology..

[60] Laderoute KR, Amin K, Calaoagan JM, Knapp M, Le T (2006). 5′-AMP-activated protein kinase (AMPK) is induced by low-oxygen and glucose deprivation conditions found in solid-tumor microenvironments.. Mol Cell Biol.

[61] Martin TL, Alquier T, Asakura K, Furukawa N, Preitner F (2006). Diet-induced obesity alters AMP kinase activity in hypothalamus and skeletal muscle.. J Biol Chem.

