# Supplementary figures and images for: AMPK Regulates Circadian Rhythms in a Tissue- and Isoform-Specific Manner
Source: PLoS One. 2011 Mar 31;6(3):e18450. doi: 10.1371/journal.pone.0018450 (PMC3069094; doi:10.1371/journal.pone.0018450)

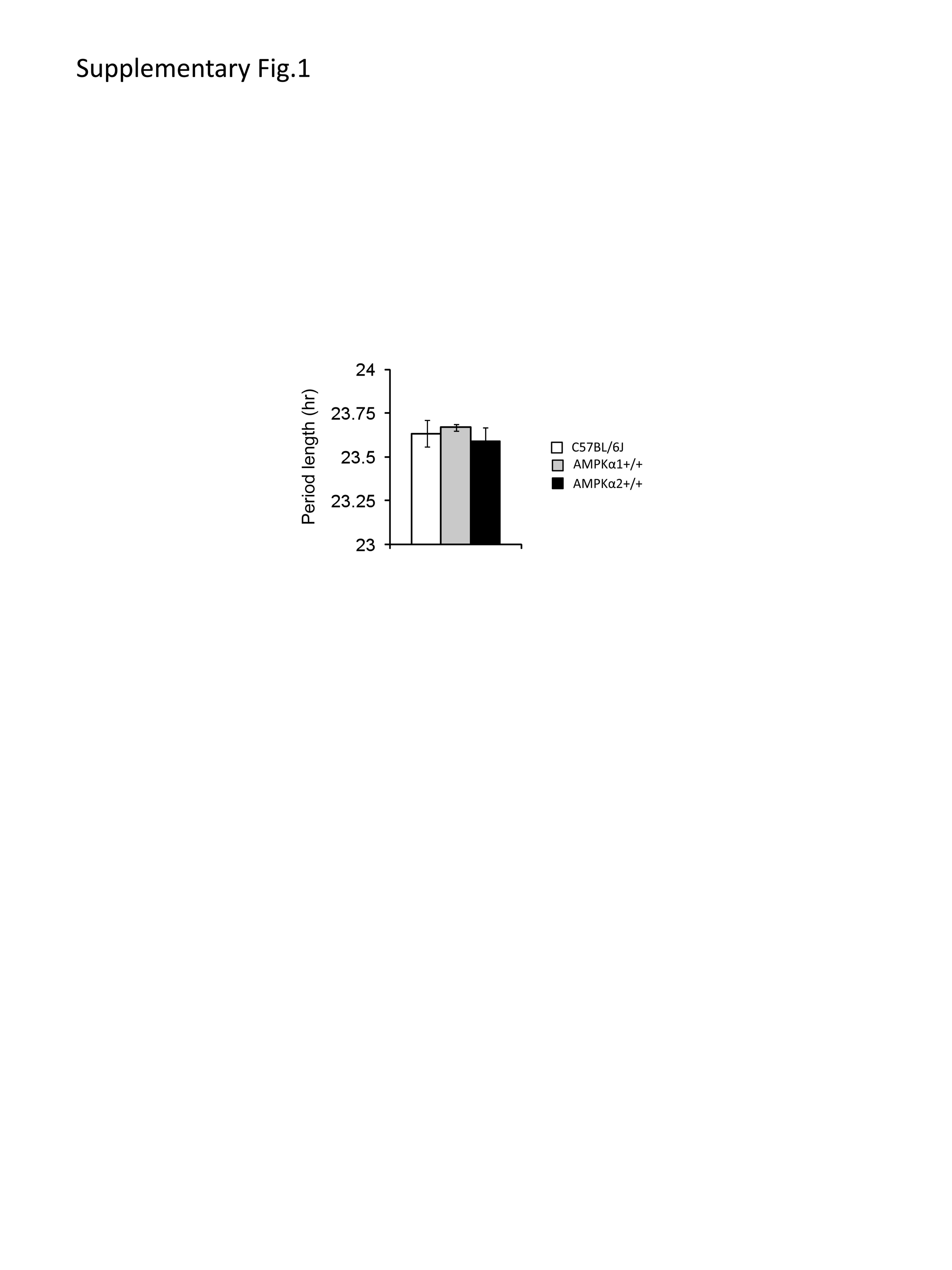

Supplement: Figure S1 — Comparison of the free-running period of wild-type controls. The free-running period of C57BL/6J (n = 5), AMPKα1+/+ (n = 5) and AMPKα2 +/+ (n = 7) mice are shown. The free-running period was determined by using the χ2 periodogram for days 1–14 in DD. Result is expressed as means ± SEM. (TIF) [file pone.0018450.s001.tif]
